# Negotiated Prices for Care at Independent and System-Affiliated Rural Hospitals

**DOI:** 10.1001/jamanetworkopen.2025.16188

**Published:** 2025-05-20

**Authors:** Cody Lendon Mullens, Mitchell Mead, James D. Lee, Janice C. Probst, Justin B. Dimick, Andrew M. Ibrahim

**Affiliations:** 1 Department of Surgery, University of Michigan, Ann Arbor; 2 UM National Clinician Scholars Program, University of Michigan, Ann Arbor; 3 Center for Healthcare Outcomes and Policy, University of Michigan, Ann Arbor; 4 University of Michigan Medical School, Ann Arbor; 5 Department of Internal Medicine, University of Michigan, Ann Arbor; 6 Arnold School of Public Health, University of South Carolina, Columbia

## Abstract

This cohort study assesses whether negotiated prices for procedural and imaging services differ at independent vs system-affiliated rural hospitals.

## Introduction

Nearly one-third of rural hospitals are at risk of closing, largely due to financial challenges.^1^ Emerging data suggest that health system affiliation is associated with higher negotiated prices for services.^2,3^ For rural hospitals, system affiliation is associated with improved financial performance, margins, and inpatient and outpatient prices broadly.^4,5,6^ However, most efforts have emphasized only critical access hospitals, a subset of rural hospitals, and have not evaluated service-specific price differences. We sought to assess whether negotiated prices for procedural and imaging services differ at independent vs system-affiliated rural hospitals.

## Methods

In this cohort study, we collected commercially negotiated prices on December 17, 2024, from Turquoise Health, a database that aggregates price data daily reported for commercial insurance plans based on publicly available data. We evaluated prices for a combination of high-margin and high-volume procedures and imaging studies that hospitals are required to report in accordance with price transparency policies. We linked negotiated prices to publicly available Healthcare Cost Report Information System (HCRIS) data from Centers for Medicare & Medicaid Services using the Hospital Identification Number to associate hospital information with price data. Using HCRIS data, we evaluated hospital characteristics and delineated rural hospitals based on US Census Designation of Core-Based Statistical Areas (CBSA). Hospitals were considered rural if they were in a rural or micropolitan CBSA. We identified independent vs system-affiliated rural hospitals by using the 2021 American Hospital Association Annual Survey.

We used 1% winsorization by service and adjusted for critical access hospital status, hospital ownership status, annual admissions, and state fixed effects to account for possible appropriate variations in negotiated prices. We used equality of proportion tests and *t* tests where appropriate when comparing hospital characteristics and prices at independent vs system-affiliated rural hospitals. All statistical tests were 2-sided, and *P* < .05 was used as our statistical significance threshold. The statistical analysis was performed from December 2024 to January 2025, using Stata Statistical Software, version 16.0. This cohort study adhered to the STROBE reporting guideline and was deemed exempt from review and informed consent by the University of Michigan institutional review board because it does not use any patient information; secondary data are used in our analysis.

## Results

There were 1063 rural hospitals (596 independent) and 426 557 negotiated prices identified. Independent and system-affiliated rural hospitals had similar bed capacity but different geographic distributions. Additionally, independent hospitals were more likely to be for profit (57.7% vs 23.1%; *P* < .001) and be a critical access hospital (80.7% vs 73.9%; *P* = .008) (Table 1). Of the 14 procedures included, 13 had higher adjusted negotiated prices at system-affiliated rural hospitals compared with their independent counterparts (Table 2). For a common procedure (eg, cholecystectomy), negotiated prices were 30.8% lower at independent rural hospitals ($5356 vs $7006; difference, $1650; *P* < .001). Similarly, all 12 imaging procedures had higher adjusted negotiated prices at system-affiliated rural hospitals (Table 2).

**Table 1.** Hospital Characteristics at Independent vs System-Affiliated Rural Hospitals

Characteristic	Hospitals, No. (%)		*P* value
	Independent	System affiliated	
No. of hospitals	596	467	
Census region			
Northeast	25 (4.2)	28 (6.0)	.18
South	208 (34.9)	179 (38.3)	.25
Midwest	234 (39.3)	217 (46.5)	.02
West	129 (21.6)	43 (9.2)	<.001
No. of beds			
1-100	199 (33.4)	151 (32.3)	.72
101-300	324 (54.4)	251 (53.7)	.84
≥301	73 (12.2)	65 (13.9)	.42
Ownership			
For profit	344 (57.7)	108 (23.1)	<.001
Not for profit	224 (37.6)	313 (67.0)	<.001
Governmental, nonfederal	28 (4.7)	46 (9.9)	.001
Critical access hospital			
No	115 (19.3)	122 (26.1)	.008
Yes	481 (80.7)	345 (73.9)	.008
Annual admissions, No.			
0-500	367 (61.6)	242 (51.8)	.001
501-1000	149 (25.0)	113 (24.2)	.76
>1000	80 (13.4)	112 (24.0)	<.001

**Table 2.** Differences in Adjusted Price-Negotiated Rates at Independent vs System-Affiliated Rural Hospitals^a^

Service	No. of negotiated prices	Price, mean (95% CI), USD		Difference (%), USD	*P* value
		Independent rural hospitals	System-affiliated rural hospitals		
Procedures					
Benign uterine and adnexa procedures	5843	16 219 (16 012-16 426)	20 441 (19 717-21 165)	4222 (26.0)	<.001
Breast excision	11 188	2688 (2650-2727)	3127 (3017-3237)	439 (16.3)	<.001
Cardiac valve procedure with cardiac catheterization	4954	125 688 (123 968-127 409)	168 378 (161 666-175 091)	42 690 (34.0)	<.001
Cataract removal	5536	3998 (3937-4060)	4833 (4660-5005)	835 (20.9)	<.001
Cervical spinal fusion	5063	30 745 (30 285-31 205)	41 735 (40 034-43 435)	10 990 (35.7)	<.001
Cholecystectomy	9689	5356 (5264-5448)	7006 (6767-7245)	1650 (30.8)	<.001
Colonoscopy	14 390	1739 (1719-1758)	2020 (1970-2071)	281 (16.2)	<.001
Diagnostic heart catheterization	4162	11 318 (11 149-11 487)	15 028 (14 235-15 820)	3710 (32.8)	<.001
Esophagogastroduodenoscopy	15 326	1588 (1569-1606)	1928 (1878-1978)	340 (21.4)	<.001
Hip or knee replacement	6834	24 981 (24 681-25 281)	29 979 (28 980-30 979)	4998 (20.0)	<.001
Inguinal hernia repair	8992	3747 (3677-3818)	4670 (4490-4850)	923 (24.6)	<.001
Noncervical spinal fusion	5568	44 777 (44 153-45 401)	57 358 (55 046-59 669)	12 581 (28.1)	<.001
Prostatectomy	2065	14 365 (14 111-14 618)	13 880 (12 711-15 049)	−485 (−3.4)	.42
Tonsil removal (patient <12 y)	4620	4148 (4044-4253)	5990 (5704-6276)	1842 (44.4)	<.001
Imaging studies					
Abdomen and pelvis CT scan with contrast	25 687	2605 (2586-2624)	2891 (2844-2938)	286 (11.0)	<.001
Abdominal ultrasound of pregnant uterus, 1st trimester	21 148	541 (537-545)	620 (610-630)	79 (14.6)	<.001
Bilateral mammography	21 288	379 (376-382)	438 (432-445)	59 (15.6)	<.001
Brain MRI with contrast	21 672	2790 (2769-2810)	3198 (3146-3249)	408 (14.6)	<.001
Complete abdominal ultrasound	22 762	654 (649-659)	735 (722-748)	81 (12.3)	<.001
4-View lower back x-ray	23 559	394 (391-398)	430 (422-438)	36 (9.0)	<.001
Head CT without contrast	24 465	1190 (1181-1199)	1282 (1259-1304)	92 (7.7)	<.001
Lower extremity MRI	43 248	1933 (1923-1944)	2291 (2263-2319)	358 (18.5)	<.001
Pelvis CT scan with contrast	22 280	1627 (1615-1639)	1805 (1775-1834)	178 (10.9)	<.001
Screening mammogram	40 687	289 (287-290)	332 (328-336)	43 (14.9)	<.001
Transvaginal ultrasound	24 589	494 (490-497)	538 (529546)	44 (8.9)	<.001
Unilateral diagnostic mammography	30 942	300 (298-302)	349 (345-354)	49 (16.5)	<.001
Total	426 557	NA	NA	NA	NA

Abbreviations: CT, computed tomography; MRI, magnetic resonance imaging; NA, not applicable; USD, US dollars.

^a^ Adjusted for critical access hospital status, hospital ownership status, annual admissions, and state fixed effects.

## Discussion

This study evaluating 426 557 negotiated prices found that independent rural hospitals had lower negotiated prices for procedural and imaging services than their system-affiliated counterparts. These data suggest 2 possible strategies to support financially struggling independent rural hospitals. First, rural hospitals could consider pursuing a health system affiliation that may give them better negotiating power and may lead to higher reimbursement, although there can be adverse consequences for system affiliation.^5^ Second, policymakers could intervene to ensure fair, equitable reimbursement for rural hospitals, especially in the context of increasing health care spending.

These findings should be interpreted in the context of their limitations. Data regarding system affiliation are from 2021, whereas pricing data are through 2024. Some hospitals may have since become affiliated, but this would bias our findings toward the null. Additionally, there may be appropriate sources of variation in negotiated prices. However, we adjusted for potential factors that may explain such variation. The rates provided may be vulnerable to reporting bias. However, compliance with these reports is federally mandated, and these data are increasingly being used to evaluate hospital prices.^2,3^

## Notes

Supplement.
Data Sharing Statement
