# Supplementary material for: Negotiated Prices for Care at Independent and System-Affiliated Rural Hospitals
Source: JAMA Netw Open. 2025 May 20;8(5):e2516188. doi: 10.1001/jamanetworkopen.2025.16188 (PMC12093185; doi:10.1001/jamanetworkopen.2025.16188)
Supplement: Supplement. — Data Sharing Statement [file jamanetwopen-e2516188-s001.pdf]

Data Sharing Statement

Mullens. Negotiated Prices for Care at Independent and System-Affiliated Rural Hospitals.
JAMA Netw Open. Published May 20, 2025. doi:10.1001/jamanetworkopen.2025.16188

Data
Data available: No

Additional Information
Explanation for why data not available: Unable to share due to data use agreements.
